# A heterozygous p.S143P mutation in *LMNA* associates with proteasome dysfunction and enhanced autophagy-mediated degradation of mutant lamins A and C

**DOI:** 10.3389/fcell.2022.932983

**Published:** 2022-08-30

**Authors:** Gun West, Minttu Turunen, Anna Aalto, Laura Virtanen, Song-Ping Li, Tiina Heliö, Annika Meinander, Pekka Taimen

**Affiliations:** ^1^ Institute of Biomedicine and FICAN West Cancer Centre, University of Turku, Turku, Finland; ^2^ InFLAMES Research Flagship Center, University of Turku and Åbo Akademi University, Turku, Finland; ^3^ Faculty of Science and Engineering, Åbo Akademi University, Turku, Finland; ^4^ Heart and Lung Center Helsinki University Hospital and University of Helsinki, Helsinki, Finland; ^5^ Department of Pathology, Laboratory Division, Turku University Hospital, Turku, Finland

**Keywords:** lamin A/C (LMNA), ubiquitin (Ub), autophagy, degradation, disease mutations, ubiquitin-proteasome degradation system

## Abstract

Lamins A and C are nuclear intermediate filament proteins that form a proteinaceous meshwork called lamina beneath the inner nuclear membrane. Mutations in the *LMNA* gene encoding lamins A and C cause a heterogenous group of inherited degenerative diseases known as laminopathies. Previous studies have revealed altered cell signaling pathways in lamin-mutant patient cells, but little is known about the fate of mutant lamins A and C within the cells. Here, we analyzed the turnover of lamins A and C in cells derived from a dilated cardiomyopathy patient with a heterozygous p.S143P mutation in *LMNA*. We found that transcriptional activation and mRNA levels of *LMNA* are increased in the primary patient fibroblasts, but the protein levels of lamins A and C remain equal in control and patient cells because of a meticulous interplay between autophagy and the ubiquitin-proteasome system (UPS). Both endogenous and ectopic expression of p.S143P lamins A and C cause significantly reduced activity of UPS and an accumulation of K48-ubiquitin chains in the nucleus. Furthermore, K48-ubiquitinated lamins A and C are degraded by compensatory enhanced autophagy, as shown by increased autophagosome formation and binding of lamins A and C to microtubule-associated protein 1A/1B-light chain 3. Finally, chaperone 4-PBA augmented protein degradation by restoring UPS activity as well as autophagy in the patient cells. In summary, our results suggest that the p.S143P-mutant lamins A and C have overloading and deleterious effects on protein degradation machinery and pharmacological interventions with compounds enhancing protein degradation may be beneficial for cell homeostasis.

## Introduction

Eukaryotic cells are continuously dealing with misfolded proteins, compromising cellular homeostasis. To maintain functional protein homeostasis, these misfolded proteins are modulated by the cellular protein quality control (PQC) systems that promote refolding, degradation, or sequestering of proteins into compartments (Arrasate et al., 2004; Powers et al., 2009). Molecular chaperones are important components of PQC and the first defense mechanism against proteotoxicity by recognizing misfolded proteins and promoting refolding (Tiroli-Cepeda and Ramos, 2011). However, if the misfolded proteins cannot be repaired, they are marked for degradation.

In eukaryotic cells, the ubiquitin-proteasome system (UPS) and the autophagy-lysosome systems are two major intracellular machineries that regulate the amount of proteins by degrading misfolded, damaged, and aggregated proteins. Under normal conditions, up to 80–90% of proteins are degraded by proteasomes that are found not only in the cytoplasm both free and attached to the endoplasmic reticulum (ER) but also throughout the nucleoplasm (Lee and Goldberg, 1998; Wójcik and DeMartino, 2003; Collins and Goldberg, 2017). Short-lived proteins are degraded through the UPS, which is initiated by sequential addition of ubiquitin chains to target proteins by the E1-E2-E3 enzyme cascade (Bachmair and Varshavsky, 1989; Hershko and Ciechanover, 1998). The 26S proteasomes are composed of the 19S regulatory particle that recognizes and unfolds the ubiquitin chains as well as the 20S core particle that hydrolyses the protein into short peptides or amino acids (Voges et al., 1999). Dysregulation of the PQC leading to aggregation of misfolded proteins is characteristic for different human diseases such as neurodegenerative diseases, cancer, diabetes, lysosomal storage diseases and cardiovascular diseases (Amm et al., 2014; Maejima, 2020).

Macroautophagy (hereafter called autophagy) is a conserved molecular pathway that accounts for 10–20% of protein degradation (Gronostajski et al., 1985). Under cellular stress conditions such as hypoxia, starvation, or DNA damage, accumulated protein aggregates and damaged organelles are increasingly eradicated through autophagy. Autophagy is mediated by double-membrane vesicles called autophagosomes that engulf cellular material to be fused with the endosomes and lysosomes for degradation (Gatica et al., 2018). Autophagy can be divided into non-selective that is a bulk degradation pathway for cytoplasmic components, and selective that specifically removes protein aggregates and damaged organelles (Glick et al., 2010). In selective autophagy, target proteins are ubiquitinated and recognized by carrier proteins such as sequestosome 1 (SQSTM1/p62), optineurin, neighbor of BRCA1, nuclear dot protein 52 kDa, Tax1-binding protein 1, and Toll-interacting protein that bring them to the autophagosomes (Lippai and Lőw, 2014). UPS and autophagy are interconnected, and inhibition of UPS has been shown to activate autophagy in a compensatory manner (Wang and Wang, 2015; Pohl and Dikic, 2019).

The nuclear lamina, a meshwork of proteins underlying the inner nuclear membrane, is primarily composed of nuclear intermediate filament proteins called lamins and their associated proteins. A-type lamins (lamins A and C) are encoded by the *LMNA* gene through alternative splicing (Lin and Worman, 1993). Lamin C is transcribed directly from the *LMNA* gene, while lamin A is expressed as a precursor (pre-lamin A) that undergoes posttranslational modification steps to become the mature lamin A (Dechat et al., 2010). The nuclear lamina provides the nucleus with mechanical strength and regulates chromatin organization and gene expression (Dahl et al., 2006; Dechat et al., 2010; Osmanagic-Myers et al., 2015; Briand and Collas, 2020). Mutations in the *LMNA* gene or alterations in its expression levels have been linked to a variety of diseases called laminopathies, and to some extent, cancer progression (Burke and Stewart, 2013; Sakthivel and Sehgal, 2016).

Previous studies have shown that a selective autophagy known as nucleophagy degrades various nuclear proteins, including lamins, during different cellular stresses (Papandreou and Tavernarakis, 2019). In support, lamins A, C and B1 have been shown to interact with microtubule-associated protein 1A/1B-light chain 3 (LC3-I/II), an autophagy-linked protein, upon DNA damage (Dou et al., 2015; Li et al., 2019). Also, cells with the p.H222P *Lmna* mutation have been shown to increasingly form autophagosomes that contain nuclear components (Park et al., 2009).

How UPS and autophagy are involved in the degradation of lamins A and C still remains poorly understood. In the current study, we investigated whether disease-associated mutations in lamins A and C alter their turnover and how functionally defective mutant lamins are processed within cells. We found that mRNA levels of lamins A and C are increased in fibroblasts obtained from a dilated cardiomyopathy patient with a heterozygous p.S143P *LMNA* mutation. However, the mutant lamins A and C are more unstable and increasingly tagged for degradation by K48-linked ubiquitination, which in turn leads to saturation of UPS. Although K48-linked ubiquitin chains are compensatorily degraded by autophagy, they still accumulate in the nucleus. A chaperone, 4-PBA, restored degradation through UPS and augmented protein degradation by further activation of autophagy. These results suggest that pharmacological intervention with compounds that enhance clearance of mutant lamin might be beneficial in some of the laminopathies.

## Materials and methods

### Cell culture

Primary fibroblasts from a healthy donor and from a patient carrying the p.S143P mutation in the *LMNA* gene were cultured in Minimum Essential Medium containing 15% fetal bovine serum, 1x penicillin/streptomycin/glutamine, 1x non-essential amino acids, and 1x vitamins (all from Invitrogen, Waltham, Massachusetts, United States) as previously described (West et al., 2016; Shah et al., 2019). HeLa cells transduced with a pFLAG-FLRU-vector expressing *LMNA* shRNA and either wild-type or p.S143P mutant lamin A insert were cultured in Dulbecco’s Modified Eagle Medium supplied with 10% fetal bovine serum and 1x penicillin/streptomycin/glutamine (all from Invitrogen) as described earlier (West et al., 2016). The use of patient derived cells was approved by the Ethics Committees of the Hospital District of Helsinki and Uusimaa (HUS 387/13/03/2009 and HUS/1187/2019). All procedures were undertaken with informed consent and according to the principles expressed in the Declaration of Helsinki.

### Chemicals and antibodies

Cells were treated with 300 μg/ml cycloheximide (CHX) (Sigma-Aldrich, St. Louis, Missouri, United States), 50 µM leptomycin B (LMB) (Santa Cruz Biotechnology, Dallas, Texas, United States), 20 mM ammonium chloride (NH_4_Cl), 1 µM MG132 (Sigma-Aldrich) and/or 5 mM 4-Phenylbutyric acid (4-PBA) (Selleck Chemicals, Houston, Texas, United States). The primary antibodies included mouse monoclonal anti-lamins A and C (1:10,000, a kind gift from Professor Robert Goldman, Northwestern University), mouse monoclonal anti-actin (1:500, clone AC-40, Sigma-Aldrich), rabbit monoclonal anti-Atg5 (1:100, clone D5F5U, #12994, Cell Signaling Technology, Danvers, Massachusetts, United States), rabbit monoclonal anti-Atg7 (1:100, clone D12B11, #8558, Cell Signaling Technology), rabbit polyclonal anti-LC3-I/II (IF:1:100, WB:1:1000, #4108, Cell Signaling Technology), mouse monoclonal anti-p62 (IF:1:100, WB:1:1000, clone 2C11, ab56416, Abcam Cambridge, United Kingdom), mouse monoclonal HRP-conjugated anti-GAPDH (1:10,000, clone 1E6D9, #HRP-60004, Proteintech, Rosemont, Illinois, United States), rabbit monoclonal anti-ubiquitin-K48 (1:400, clone Apu2, #ZRB2150, Sigma-Aldrich) and mouse monoclonal vimentin (1:100, clone V6, #V6630, Sigma-Aldrich). Secondary antibodies for western blotting were HRP-conjugated donkey anti-rabbit-IgG and sheep anti-mouse-IgG (both from Thermo Fischer Scientific, Waltham, Massachusetts, United States). Secondary antibodies for immunofluorescence were Alexa 488/555 goat-anti-mouse and goat-anti-rabbit antibodies (all from Thermo Fischer Scientific). Pan-ubiquitin chains were purified using a protein consisting of four ubiquitin-associated domains in tandem fused to GST and His (referred to as pan-TUBE), kindly provided by Professor Mads Gyrd Hansen, University of Copenhagen, Denmark.

### Reverse transcription quantitative polymerase chain reaction (RT-qPCR)

RNA was extracted using the NucleoSpin RNA kit (Macherey-Nagel, Düren, Germany) and 1 µg of high-quality RNA was converted to cDNA using the SensiFAST cDNA synthesis kit (Bioline, Toronto, Ontario, Canada). Amplification was performed using a SensiFAST SYBR Lo-ROX PCR kit (Bioline) for 3 min at 95°C followed by 40 cycles of 5 s at 95°C, 10 s at 60°C and 15 s at 72°C. The fold change was calculated using the 2−ΔΔCt method and normalized to GAPDH. The primers used were for GAPDH 5′-TAA​ATT​GAG​CCC​GCA​GCC​TCC​C-3′ and 5′-ATG​TGG​CTC​GGC​TGG​CGA​CG-3’; *LMNA* total 5′-GGG​ATG​CCC​GCA​AGA​CCC​TT-3′ and 5′-GGT​ATT​GCG​CGC​TTT​CAG​CTC​C-3’; *LMNA* WT 5′-GCT​CTG​CTG​AAC​TCC​AAG​GAG​G-3′ and 5′-GCC​TCA​AGC​TTG​GCC​ACC​TG-3’; *LMNA* S143P 5′-GCT​CTG​CTG​AAC​CCC​AAG​GAG​G-3′ and 5′-GCC​TCA​AGC​TTG​GCC​ACC​TG-3’.

### Mass spectrometry

The mass spectrometry analyses were performed at the Turku Proteomics Facility as follows: proteins separated on an SDS-PAGE gel were digested with peptides dissolved in 15 μL of 0.1% formic acid. The Liquid Chromatography Electrospray Ionization Tandem Mass Spectrometric (LC-ESI-MS/MS) analyses were performed on a nanoflow HPLC system (Easy-nLC1200, Thermo Fisher Scientific) coupled to the Orbitrap Fusion Lumos Tribrid mass spectrometer (Thermo Fisher Scientific) equipped with a nano-electrospray ionization source. Peptides were first loaded on a trapping column and subsequently separated inline on a 15 cm C18 column (75 μm × 15 cm, ReproSil- Pur 5 μm 200 A C18-AQ, Dr. Maisch HPLC GmbH, Ammerbuch-Entringen, Germany). The mobile phase consisted of water with 0.1% formic acid (solvent A) or acetonitrile/water (80:20 (v/v)) with 0.1% formic acid (solvent B). A 30 min gradient from 8 to 37% B was used to elute peptides. Mass spectrometry data were acquired automatically by using Thermo Xcalibur 4.1 software (Thermo Fisher Scientific). An information-dependent acquisition method consisted of an Orbitrap MS survey scan of the mass range 300–1300 m/z followed by HCD fragmentation in a top speed mode with a 3 s cycle time for precursor selection. An inclusion list including possible tryptic peptides containing the mutation p.S143P site was created and added to the LC-MS/MS method.

### Luciferase assay of *LMNA* promoter activity

The human promoter region of *LMNA* was recognized from the earlier published rat *LMNA* promoter sequence (Tiwari et al., 1998). The promoter sequence was chosen to include the TATA box, GC box, and activator protein 1 (AP1) binding site. The upstream -1.3 kB promoter region (sequence -1334 to-16) was synthesized and subcloned into a pNLCoI1 [luc2-P2A-NlucP/Hygro] vector (Promega, Madison, Wisconsin, United States) at GenScript, Netherlands. The vector (50 ng/ml) was transfected into fibroblasts using Lipofectamine (Thermo Fischer Scientific) and, after 72 h, Dual Glo luciferase assay reagent was added. After an incubation time of 15 min at RT, the firefly luciferase was measured on a luminometer. Then Dual Glo Stop and Go reagent was added, incubated for 15 min, and renilla fluorescence was measured on a luminometer. The background was subtracted and the values were normalized to renilla (firefly/renilla).

### Western blot analysis

Cells were pelleted, washed twice with 1xPBS at 4°C, and solubilized in M-PER mammalian protein extraction reagent (Thermo Fisher Scientific) supplemented with 1x protease and 1x phosphatase inhibitors. Whole cell extract was boiled in Laemmli buffer and separated on a 4–10% gradient gel (BioRad, Hercules, California, United States), transferred to a nitrocellulose membrane (BioRad) and incubated for 1 h in RT or overnight at 4°C with primary antibody. The membrane was incubated with horseradish peroxidase (HRP)-conjugated secondary antibody (1:10,000) for 1 h at RT. The chemiluminescent signal was detected with an Enhanced Chemiluminescence kit (Thermo Fischer Scientific) using a ChemiDoc MP (BioRad). The blots were quantified with ImageJ and their values normalized by dividing them with GAPDH values. The western blots were repeated independently at least two times.

### Immunofluorescence and confocal microscopy

Cells grown on glass plates were fixed with 10% formalin for 10 min, followed by permeabilization with 0.1% Triton-X for 10 min. Samples were incubated with primary antibodies for 1 h at RT followed by secondary antibodies (1:400) for 1 h at RT. LysoTracker Red DND-99 (Thermo Fischer) was added to cells at a concentration of 100 nM and incubated for 30 min. Acridine orange (Invitrogen, 3568) was diluted in PBS and used at a final concentration of 10 μg/ml on fixed and permeabilized cells for 15 min. All immunofluorescence glass plates were mounted with ProLong Diamond Antifade Mountant, including DAPI (Thermo Fischer Scientific). Confocal images were taken on a Marianas 3i Yokogawa CSU-W1 spinning disk confocal microscope attached to an inverted Zeiss AxioObserver Z1 microscope (Intelligent Imaging Innovations GmbH, Göttingen, Germany). The microscope was controlled by SlideBook six software (Intelligent Imaging Innovations GmbH) and a 63x/1.4 Zeiss Plan-Apochromat oil objective was used. The mean fluorescence intensities were analyzed with ImageJ (National Institutes of Health) or Fiji software (Schindelin et al., 2012) using 20x mid-plane sections of confocal images or images taken with a Nikon Eclipse Ni microscope with a 20x/0.5 Plan-Apochromat objective. The Pearson’s correlation coefficients were determined from 10 confocal images (60–100 individual cells) taken with 63× objective and analyzed with the coloc2 plugin in Fiji.

### Proximity ligation assay

Cells grown on glass plates were fixed with 10% formalin for 10 min and permeabilized with 0.1% Triton X-100 in 1xPBS for 10 min. Duolink PLA kit (DUO92105, Millipore Sigma, Burlington, Massachusetts, United States) was used according to the manufacturer’s protocol. After blocking, the samples were incubated with primary antibodies in a humified chamber at 37°C for 1 h, followed by incubation with secondary antibodies (anti-rabbit PLUS and anti-mouse MINUS PLA-probes). Following the ligation and amplification steps, the coverslips were mounted with ProLong Diamond with DAPI. The number of PLA signals per cell was determined visually, and the cells with more than three PLA signals were considered positive.

### Proteasome purification and activity assay

A proteasome 20S activity assay kit (MAK172, Millipore Sigma) was used for live-cell measurements of proteasome activity in patient and control fibroblasts (80.000 cells per well on a 96-well plate). Any inhibitors were added 24 h prior to measurements, and the proteasome assay loading solution 1 h before measurements. A plate reader at 490/525 nm (excitation/emission) was used for detection.

To isolate 20S proteasomes from control and patient fibroblasts or from lentivirally transduced HeLa cells expressing either FLAG-tagged WT-lamin A or p.S143P lamin A, the cells were first washed three times with cold 1xPBS and further lysed with a buffer containing 50 mM HEPES pH 7.5, 5 mM EDTA, 150 mM NaCl, and 1% Triton-X100 for 30 min on ice. The cell lysates were centrifuged at 15,000 rpm for 15 min at 4°C, and the supernatants were analyzed with a 20S proteasome assay kit (Bio-Techne Ltd, Abingdon, United Kingdom) according to the manufacturer’s protocol. Briefly, the proteasomes were activated in a 1x reaction buffer containing sodium dodecyl sulfate (SDS) and incubated for 20 min at 37°C before being mixed with the fluorogenic peptide suc-LLVY-AMC. The amount of cleaved AMC fragment was measured using a plate reader at 345/445 nm (excitation/emission). Any inhibitors were added 24 h prior to harvesting the cells, and the same inhibitors were used when adding the fluorogenic substrate. The background was subtracted and the values from control cells were used to normalize the results.

### Purification of ubiquitin conjugates from cells under denaturing conditions

An equal number of cells were washed twice with 1xPBS and pelleted before lysing using a buffer containing 50 mM Tris pH 7.5, 150 mM NaCl, 1% Triton X-100, 1 mM EDTA, 10% glycerol supplemented with 1 mM DTT, 5 mM NEM, 1x protease and phosphatase inhibitors, 5 mM chloroacetamide, and 1% SDS. Lysates were sonicated, diluted to 0.1% SDS, and cleared before incubation with Glutathione Sepharose™ 4B (BioRad). For purification with the recombinant protein GST-TUBE (30–100 mg/ml), the lysate was incubated with the beads for a minimum of 2 h under rotation at 4°C. The beads were washed three times with ice cold wash buffer containing 10 mM Tris pH 7.5, 150 mM NaCl, 0.1% Triton X-100, 5% glycerol, and eluted using Laemmli sample buffer. The samples were boiled for 10 min and separated on a 4–10% gel.

For purification with antibody, the beads were first incubated with the lysate under rotation at 4°C overnight. After adding 1 µg of antibody and 1 h of incubation, the beads were washed twice with 1xPBS and the samples were dissolved in Laemmli buffer. The samples were boiled for 10 min and separated on a 4–10% gel.

## Results

### Lamins A and C are upregulated at mRNA, but not at protein level in *LMNA*-mutant patient cells

**FIGURE 1:**
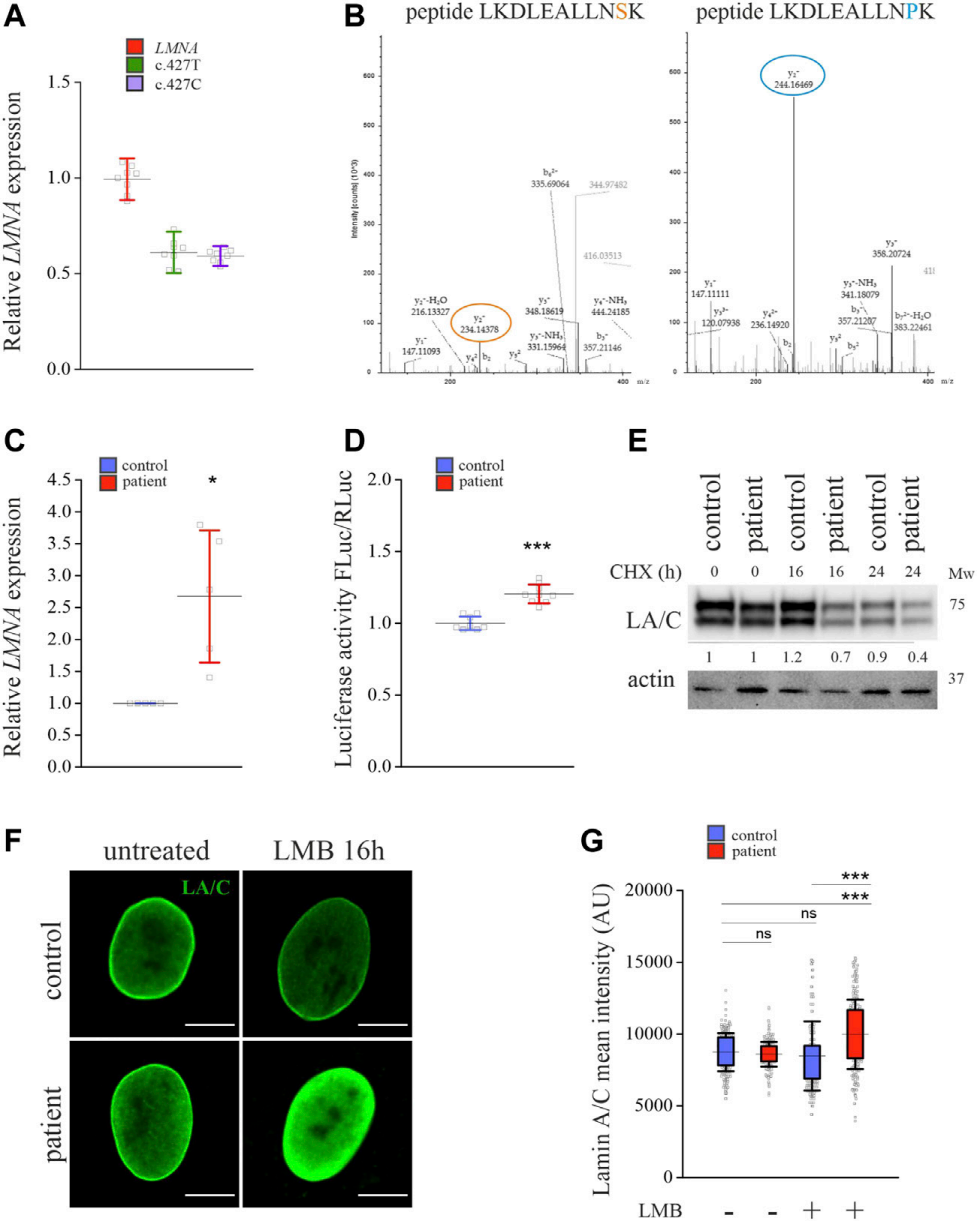
Expression and degradation of lamins A and C are enhanced in *LMNA* mutant patient cells. **(A)** RT-qPCR analysis of *LMNA* expression in the patient fibroblasts carrying a heterozygous p.S143P *LMNA* mutation. Allele-specific primers detecting either wild-type (c.427T) or mutant (c.427C) alleles, or primers detecting both (*LMNA*) were used. N = 3 individual experiments. **(B)** LC-ESI-MS/MS analysis of peptide fragments covering both wild-type and mutant lamins A and C in the patient cells. The sequences specific to the p.143S and p.143P lamin A and C peptide fragments are shown on the top, and the corresponding peaks in the mass spectra are encircled. **(C)** RT-qPCR analysis of relative overall *LMNA* expression in the control and patient fibroblasts (N = 3). **(D)** Luciferase assay measuring activity of an upstream −1.3 kb *LMNA* promotor sequence in the control and patient fibroblasts (N = 3). **(E)** Western blot analysis shows protein levels of lamins A and C (LA/C) in the patient and control fibroblasts treated with 300 μg/ml cycloheximide (CHX) for given time points. Numerical values show levels of lamins A and C normalized to actin, which was used as a loading control. **(F)** Confocal microscopy images from control and patient cells stained for lamins A and C before or after 16-h treatment with 50 µM leptomycin B (LMB). Mid-plane confocal sections are shown. Scale bar 10 µm. **(G)** Mean fluorescence intensities (AU) were measured from confocal images and plotted (N = 300). Note upregulation of lamins A and C after 16-h treatment with 50 µM LMB in the patient fibroblasts. The whiskers show the mean values ±s.d. and boxplots show the 75th and 25th percentiles of the calculated intensities, *p < 0.05, **p < 0.01, ***p < 0.001.

To study whether a disease-related point mutation in lamins A and C affect their turnover, we took advantage of primary fibroblasts obtained from a patient carrying a heterozygous p.S143P (c.427C) missense mutation in *LMNA*. Based on RT-qPCR, equal amounts of *LMNA* mRNAs with both c.427T and c.427C sequences were produced in the patient fibroblasts, indicating that both the wild-type (WT) and the mutant allele are transcribed in a similar manner (Figure 1A). Similarly, mass spectrometry analysis detected both WT and p.S143P specific protein fragments of lamins A and C in the patient cells, suggesting that both transcripts are translated into proteins (Figure 1B). However, total *LMNA* mRNA levels were significantly higher in the patient cells when compared to control cells from a healthy individual (Figure 1C). This was supported by a luciferase assay that showed higher *LMNA* promotor activity in the patient cells compared to controls (Figure 1D). Despite the increased transcription, the protein levels of lamins A and C in the patient cells were similar to controls under normal culture conditions (Figure 1E). This prompted us to analyze the stability of the lamins A and C upon inhibition of protein synthesis with cycloheximide. After 16- and 24-h treatments with cycloheximide, we noted an accelerated reduction of lamin A and C protein levels in the patient cells, indicating that they are more unstable and potentially increasingly degraded (Figure 1E).

We further asked whether lamins A and C in the patient cells are properly transported into the nucleus and treated the cells with leptomycin B, which inhibits nuclear export by blocking binding of chromosomal regional maintenance protein (CRM1) to leucine-rich nuclear export signals (Kudo et al., 1999). Calculated mean fluorescence intensity after 16-h leptomycin B treatment showed increased accumulation of lamins A and C in the nucleus of patient cells, suggesting that they are transported into nuclei and that nuclear export by CRM1 is required for their turnover (Figures 1F,G). Taken together, the results indicate that lamins A and C are increasingly produced in the patient cells, accumulate in the nucleus, and are further transported into the cytoplasm for degradation.

### Lamins A and C are increasingly ubiquitinated by K48-linked chains in *LMNA*-mutant patient cells

**FIGURE 2:**
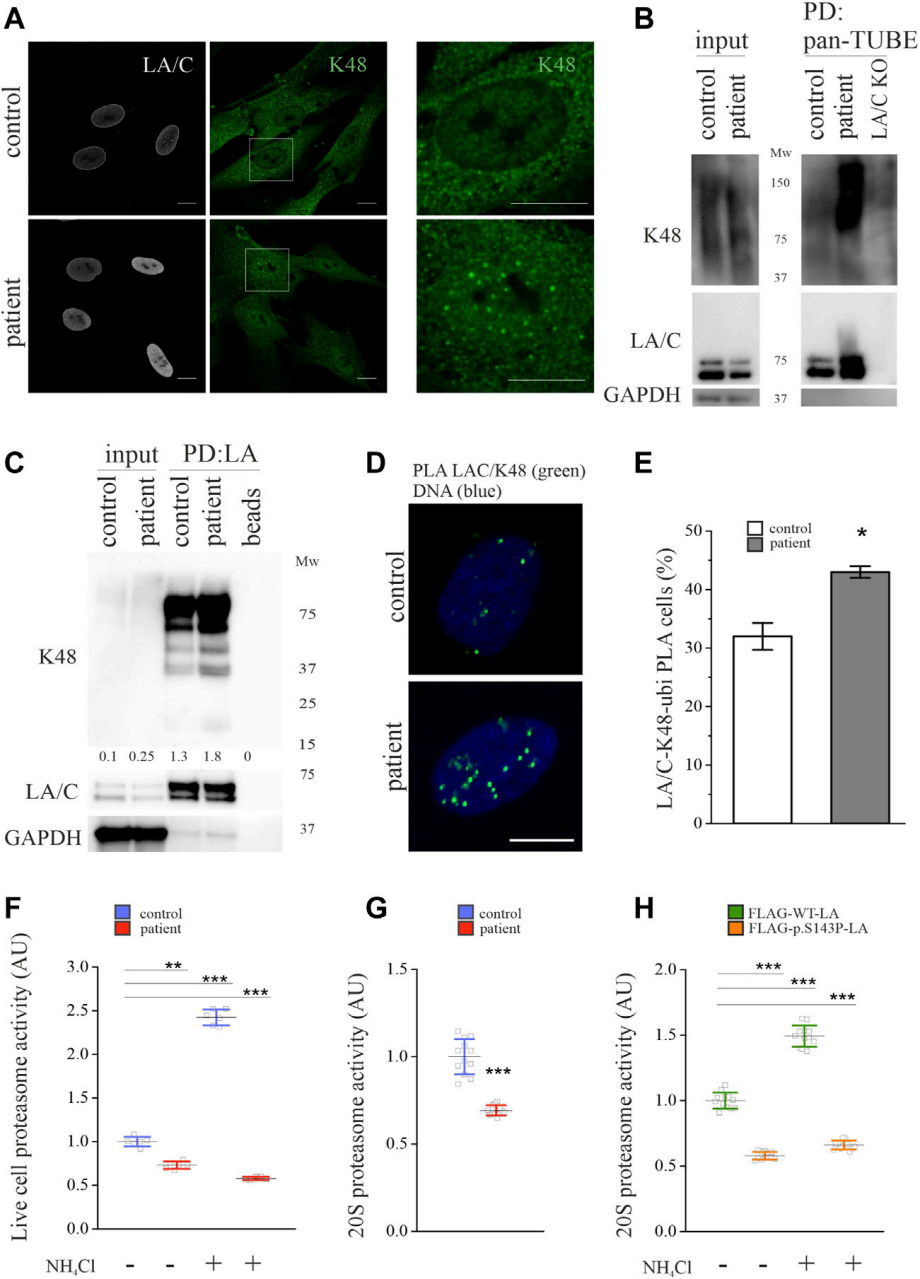
Lamins A and C are ubiquitinated by K48-linked chains in *LMNA* mutant patient fibroblasts. **(A)** Representative confocal microscopy images from control and patient fibroblasts stained for lamins A and C and K48-linked ubiquitin chains. Scale bar 10 µm. **(B)** Pulldown with GST-tagged recombinant pan-tandem ubiquitin-binding entity (pan-TUBE) under denatured conditions. **(C)** Pulldown with lamin A antibody under denatured conditions. K48-ubiquitin levels were normalized to GAPDH, which was used as a loading control. **(D-E)** Proximity ligation assay (PLA) of lamins A and C and K48-ubiquitin in the control and patient fibroblasts as calculated from >300 individual cells. Cells with more than three PLA signals were considered positive. Data are expressed as mean ± s.e.m, *p < 0.05. **(F)** Proteasome activity of control and patient fibroblasts treated with or without 20 mM NH_4_Cl for 24 h. N = 3 individual experiments. **(G)** Chymotrypsin-like activity of 20S proteasomes isolated from control and patient fibroblasts (N = 3). **(H)** Chymotrypsin-like activity of 20S proteasomes isolated from Hela cells expressing either FLAG-tagged WT-LA or p.S143P-LA and treated with or without 20 mM NH_4_Cl for 24 h (N = 3). The whiskers show mean values ±s.d **p < 0.01, ***p < 0.001.

We next asked if lamins A and C were increasingly K48-ubiquitinated in the patient cells compared to control cells. Ubiquitin contains seven lysine residues (K6, K11, K27, K29, K33, K48, K63) and one methionine (M1) that can attach to other ubiquitin chains, forming linear or branched linkages (Pohl and Dikic, 2019). Ubiquitination is mediated by the attachment of ubiquitin chains of different linkages and sizes to the lysine residues of target proteins. Among the eight different ubiquitin linkages, the lysine 48 (K48)-linkage is the most studied and is a canonical signal for protein degradation (Chau et al., 1989). Other ubiquitin linkages such as K6, K11, K27, and K29 have also been shown to mediate proteasomal degradation, whereas K48 and K63 are also involved in lysosomal degradation (Dammer et al., 2011). The K48-ubiquitin staining was predominantly nuclear in the patient fibroblasts, whereas a predominantly cytoplasmic staining pattern was observed in the control cells (Figure 2A). To analyze if lamins A and C were modified by ubiquitination, we used a GST-tagged recombinant pan-tandem ubiquitin-binding entity (pan-TUBE) to pull down ubiquitinated substrates under denatured conditions. Coherent with the K48-staining, there was more overall K48-ubiquitin in the patient cells compared to control cells (Figure 2B). The pulldown showed that lamins A and C were ubiquitinated in both cell cultures, but increasingly in the patient cells (Figure 2B). Additionally, a smear denoting ubiquitinated lamins A and C with higher molecular weight was noted in the patient cells. Likewise, immunoprecipitation with the lamin A antibody verified that lamins A and C are increasingly K48-ubiquitinated in the patient cells compared to controls (Figure 2C). The association between lamins A and C and K48-ubiquitin was further studied with PLA, which detected a statistically significantly increased number of PLA signals in the patient cells relative to controls (Figures 2D,E). These results strongly argue that lamins A and C are increasingly ubiquitinated with K48-linked chains in the patient cells.

### Degradation through the ubiquitin-proteasome system is impaired in the patient cells

The ubiquitin-proteasome system (UPS) and autophagy are the two major degradation pathways in eukaryotic cells. To find out which mechanisms are responsible for the degradation of lamins A and C in the patient and control cells, we first analyzed the rate of UPS-mediated degradation in the patient cells compared to control cells. The 20S proteasome has seven β-subunits connected to the proteolytic activity of UPS. The β-subunits possess three different enzyme specificities, namely caspase-like, trypsin-like and chymotrypsin-like activity, whereof the chymotrypsin-like activity is widely assumed to reflect the degree of protein degradation (Kisselev et al., 2003). Therefore, the proteasome activity was analyzed on live cells (Figure 2F) and on isolated 20S proteasomes (Figure 2G) by using a fluorogenic substrate specific for chymotrypsin-like activity. Interestingly, we detected only a ∼70% overall chymotrypsin-like activity in the patient cells compared to controls (Figure 2F). In an analogue, the chymotrypsin-like activity of isolated 20S proteasomes was decreased in the patient cells compared to controls (Figure 2G). Since inhibition or impairment of UPS or autophagy leads to compensatory activation of the other system, we treated the cells with ammonium chloride (NH_4_Cl) to shut down lysosomal degradation and therefore the autophagy pathway. The chymotrypsin-like activity increased in control cells after 24-h NH_4_Cl treatment, while it had no positive effect on the patient cells, indicating dysfunctional proteasome activity (Figure 2F). The impact of the p.S143P mutant lamin was further verified by measuring chymotrypsin-like activity in lentivirally transduced HeLa cells expressing either FLAG-tagged wild-type lamin A (WT-LA) or p.S143P mutant lamin A (p.S143P-LA) (Figure 2H). Correspondingly, the chymotrypsin-like activity was 50% lower in FLAG-p.S143P-LA expressing cells compared to FLAG-WT-LA expressing cells, indicating that the expression of mutant lamin leads to reduced proteasome activity (Figure 2H). Furthermore, 24-h NH_4_Cl treatment increased the chymotrypsin-like activity of FLAG-WT-LA expressing cells but had no significant effect on FLAG-p.S143P-LA expressing cells (Figure 2H). In conclusion, the results show that UPS activity is decreased in the patient cells and unresponsive to inhibition of autophagy.

### Degradation of lamins A and C through autophagy is enhanced in *LMNA*-mutant patient cells

**FIGURE 3:**
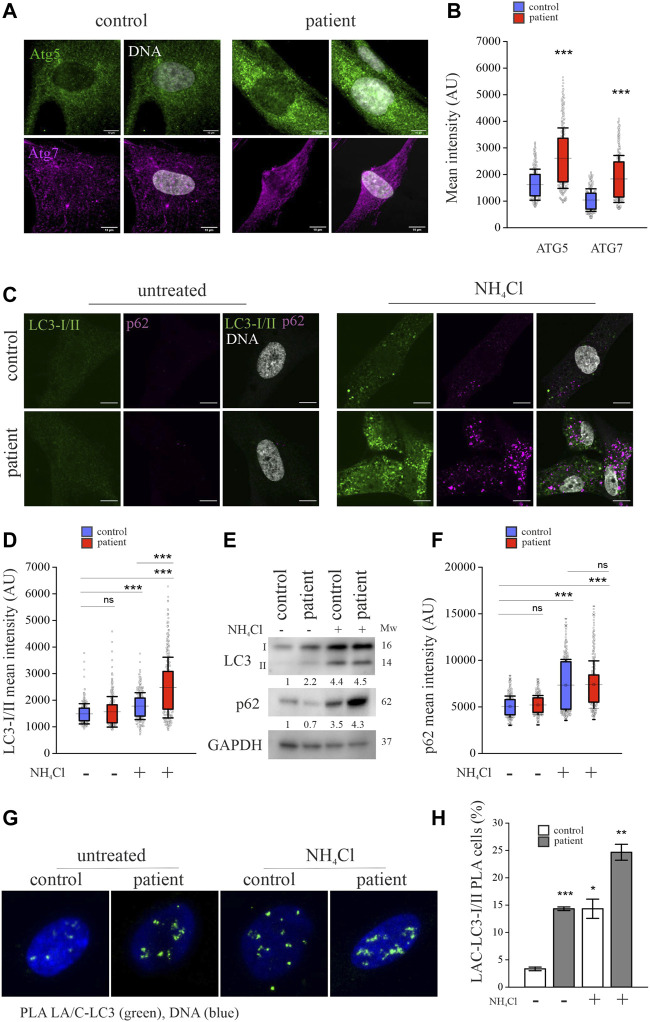
Lamins A and C are increasingly committed to autophagy in *LMNA* mutant patient fibroblasts. **(A)** Confocal microscopy images from the patient and control fibroblasts stained for autophagy-related proteins Atg5 and Atg7. Scale bar 10 µm. **(B)** Calculated mean fluorescence intensity values from Atg5 and Atg7 stainings. The whiskers show mean values ± s.d and boxplots show the 75th and 25th percentiles (N = 300), *** *p* < 0.001. **(C)** Confocal microscopy images from the patient and control fibroblasts treated with or without 20 mM NH_4_Cl for 24h and stained for Atg8/LC3-I/II and SQSTM1/p62. Note the accumulation of LC3-I/II or p62 after NH_4_Cl treatment especially in the patient cells. Scale bar 10 µm. **(D)** Calculated mean fluorescence intensity values from LC3-I/II staining. The whiskers show mean values ± s.d and boxplots show the 75th and 25th percentiles (N = 300), *** *p* < 0.001 (compared to untreated control cells). **(E)** Western blot analysis from control and patient fibroblasts treated with 20 mM NH_4_Cl for 24 h. Pooled LC3-I/II and p62 levels were normalized to GAPDH, which was used as a loading control. Note the increase of LC3-II after NH_4_Cl treatment. **(F)** Calculated mean fluorescence intensity values from p62 staining. (N = 300), *** *p* < 0.001 **(G–H)** Proximity ligation assay (PLA) detecting association of lamins A and C with LC3-I/II in the control and patient fibroblasts. The percentage of cells with PLA signals was determined from >300 cells and the cells with more than three PLA signals were considered positive. Data is expressed as mean ± s.e.m, * *p* < 0.05, ** *p* < 0.01, *** *p* < 0.001.

To find out whether lamins A and C are degraded by autophagy, we focused on ATG5, ATG7, ATG8/LC3-I/II and SQSTM1/p62, which are critical in turnover by autophagy (Komatsu and Ichimura, 2010; Martens and Fracchiolla, 2020; Collier et al., 2021). Confocal microscopy showed increased ATG5 and ATG7 staining intensity in the perinuclear area of the patient cells and this finding was verified with calculated mean fluorescence intensity values from microscopy images, indicating increased nascent autophagosome formation in these cells (Figures 3A,B). Conversion of LC3-1 to LC3-II is generated by the conjugation of cytosolic LC3-I to phosphatidylethanolamine on the surface of nascent autophagosomes. Staining for LC3-I/II showed very little or no difference between the patient and control cells under normal culture conditions (Figure 3C). However, cytoplasmic LC3-I/II accumulation was pronounced in the patient cells after inhibiting autophagosome fusion to lysosomes by NH_4_Cl treatment (Kawai et al., 2007; Yu et al., 2013; Teves et al., 2017) (Figure 3C). Similarly, calculated mean fluorescence intensities of LC3-I/II were higher in the patient cells after NH_4_Cl treatment (Figure 3D). Western blot analysis confirmed that NH_4_Cl treatment increased LC3 levels due to accumulation of LC3-II and also indicated that the autophagy flux is upregulated in patient cells (Figure 3E). SQSTM1/p62, an autophagic transport protein, binds ubiquitinated proteins and mediates their degradation by directly binding to LC3-II (Lippai and Lőw, 2014). Confocal images visualized elevated levels of p62 after inhibiting autophagy with NH_4_Cl in both the control and patient cells (Figure 3C), showing that p62 is an autophagy substrate, as previously shown by others (Pankiv et al., 2007). Similar to LC3, calculated mean fluorescence intensities and western blot analysis confirmed increased levels of p62, especially in the patient cells after autophagy inhibition (Figures 3E,F). Correlation analysis showed co-localization of p62 and LC3-I/II in control and patient cells under normal cell culture conditions, and NH_4_Cl treatment further increased the co-localization, indicating spatial accumulation of both proteins due to autophagy inhibition (Supplementary Figures S1A). NH_4_Cl treatment, however, had no detectable effect on co-localization of p62 and lamins A and C (Supplementary Figures S1B-C).

To further examine the role of autophagy in the degradation of lamins A and C, we used a proximity ligation assay (PLA) to confirm the association between lamins A and C and LC3-I/II. PLA signals were seen in 15% of patient cells compared to 4% of control cells under normal culture conditions, and their prevalence further increased after NH_4_Cl treatment (Figures 3G,H). These results suggest that lamins A and C bind to LC3 and are degraded through autophagy in both cell cultures, but the autophagic flux is increased in the patient cells.

### Crosstalk between autophagy and UPS is impaired in *LMNA*-mutant patient cells

**FIGURE 4:**
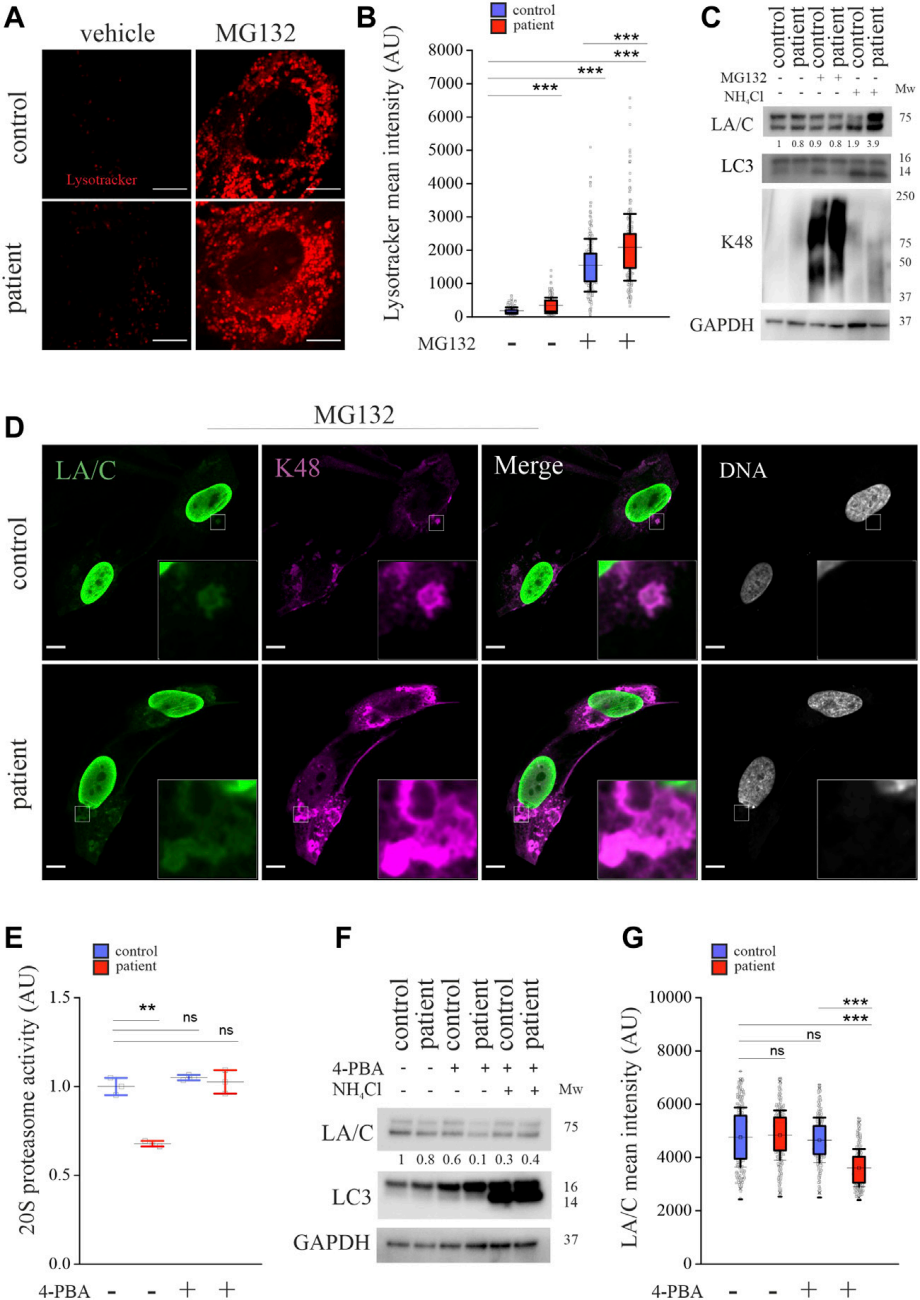
Crosstalk between autophagy and UPS is impaired in *LMNA* mutant patient cells. **(A)** LysoTracker Red DND-99 staining from untreated and MG132-treated control and patient cells. **(B)** Calculated mean fluorescence intensity values show more LysoTracker positive lysosomes in the patient cells and their number is further increased after 24-h treatment with 1 µM MG132. **(C)** Western blot analysis from control and patient cells treated with 1 µM MG132 or 20 mM NH_4_Cl for 24 h. Protein levels of lamins A and C (LA/C) were normalized to GAPDH, which was used as a loading control. All the proteins were detected on the same membrane. **(D)** Confocal microscopy images from control and patient cells treated with MG132 and stained for lamins A and C and K48-ubiquitin. Insets show cytosolic structures that co-stain with lamins A and C and K48 antibodies. Scale bar 10 µm. **(E)** Chymotrypsin-like activity as measured in control and patient cells treated with 5 mM 4-PBA for 24 h (N = 3 individual experiments) **p < 0.01. **(F)** Western blot analysis of control and patient cells treated with or without 5 mM 4-PBA. Autophagy was inhibited with 20 mM NH_4_Cl and GAPDH was used as a loading control. **(G)** Calculated mean fluorescence intensity values (AU) from control and patient fibroblasts treated with or without 5 mM 4-PBA for 24 h and stained for lamins A and C (N = 300). The whiskers show mean values ±s.d and boxplots show the 75th and 25th percentiles, *p < 0.05, ***p < 0.001.

Previous studies have shown that UPS inhibition can activate autophagy (Albornoz et al., 2019). To test this, we stained the cells with LysoTracker Red, which is a marker for acidic organelles, including lysosomes. The confocal images and calculated mean fluorescence intensities showed more LysoTracker Red positive acidic organelles in the patient cells and their number further increased upon MG132 treatment (Figures 4A,B). We also stained the cells with another marker, acridine orange (AO), which is a stain that fluoresces green in the cytoplasm or when bound to DNA, whereas AO trapped in acidic vesicular organelles or bound to RNA fluoresces red. We noticed more AO-positive red vesicular structures in the patient cells’ cytoplasm and their number further increased upon MG132 treatment, indicating either elevated formation or slowed processing of acidic organelles in these cells (Supplementary Figures S2A). Autophagy flux was further analyzed with western blot analysis showing increased LC3-II after NH_4_Cl treatment in both cell cultures, indicating inhibition of autophagy (Figure 4C). MG132 treatment showed a slight increase in LC3-II in the control cells but no change in the patient cells. Taken together, the results indicate that autophagy is activated in the patient cells after MG132 treatment. As expected, K48-ubiquitin chains were enriched after MG132 treatment in both cell cultures, but there was also a detectable increase in the patient cells after NH_4_Cl treatment (Figure 4C). MG132 treatment had no effect on protein levels of lamins A and C in either of the cell cultures (Figure 4C). However, NH_4_Cl treatment increased lamin A and C levels, especially in the patient cells, verifying our results that lamins A and C are continuously degraded through an autophagy/lysosomal pathway in these cells (Figure 4C). To analyze the fate of K48-linked lamins A and C, the cells were stained for K48-ubiquitin, and lamins A and C after MG132 treatment. Interestingly, we noticed an accumulation of cytoplasmic lamin A and C positive particles that co-stained with K48-ubiquitin especially in the patient cells (Figure 4D). This structure was rarely found in the control cells and was negative in DNA staining, excluding the possibility of nuclear leakage. The Pearson’s correlation coefficients of K48-ubiquitin and lamin A and C stainings were 0.41 and 0.44 for control and patient cells, respectively. Further staining for vimentin showed that K48 accumulations in the cytoplasm were surrounded by vimentin cages (Supplementary Figures S2B), as previously reported for aggresomes (Johnston et al., 1998). Taken together, the results suggest that K48-tagged lamins A and C are degraded by UPS but in the patient cells also through autophagy in a compensatory manner upon UPS dysfunction.

4-Phenylbutyric acid (4-PBA) is a chaperone that binds to hydrophobic parts of unfolded proteins and protects cells from protein aggregation, promotes proteins folding and reduces ER stress (Iannitti and Palmieri, 2011). Similarly, we have previously shown that 4-PBA alleviates aggregation of mutant lamins A and C and ER stress in p.S143P primary cells (West et al., 2016). To study whether 4-PBA could be used to restore the UPS activity in the patient cells, the cells were treated with 4-PBA for 24 h. Chymotrypsin-like activity was rescued in the patient cells while no additive effect was noted in the control cells (Figure 4E). Previous studies have shown that 4-PBA also enhances autophagy (Nissar et al., 2017; Gadallah et al., 2021), and correspondingly, we detected increased LC3-I levels in the patient and control cells after 4-PBA treatment (Figure 4F). When 4-PBA was combined with NH_4_Cl, LC3-II levels increased significantly, verifying that autophagy flux is enhanced under these circumstances (Figure 4F). Protein levels of lamins A and C slightly decreased after 4-PBA treatment in the patient cells, as seen by western blot and calculated mean fluorescence intensity values from cells stained for lamins A and C (Figures 4F,G). Furthermore, lamin A and C protein levels remained low when patient cells were treated with both 4-PBA and NH_4_Cl, suggesting that UPS-mediated degradation of lamins A and C was restored. Taken together, the results show that UPS dysfunction in the patient cells can be reversed by 4-PBA treatment.

## Discussion

Mutations in the *LMNA* gene cause disorders with a wide variety of clinical phenotypes. The most common laminopathy is dilated cardiomyopathy, which is almost exclusively due to heterozygous missense mutations of the *LMNA*. In the current study, we asked whether the dilated cardiomyopathy-associated p.S143P mutation in *LMNA* affects turnover and processing of lamins A and C. We found that transcriptional activity and mRNA levels of lamins A and C are increased in patient fibroblasts while their protein levels remain similar to controls. This finding in particular raised the question about the fate of mutant lamins and prompted us to focus on UPS and autophagy, the main systems upholding cellular homeostasis by protein degradation.

We show that a fraction of lamins A and C in the patient cells are short-lived, increasingly K48-ubiquitinated and targeted for degradation by UPS. However, the function of UPS, as measured by chymotrypsin-like activity, is reduced in the patient cells, which leads to an accumulation of K48-linked ubiquitin chains within the nucleus. UPS has a critical role in removing misfolded, damaged and mutated proteins, and several studies have reported accumulation of ubiquitinated protein targets, protein aggregates, and UPS impairment in different heart diseases (Gilda and Gomes, 2017). Previously, Cattin et al. showed that heterozygous Lmna^ΔK32/+^ mice develop dilated cardiomyopathy and have increased degradation of ΔK32-lamins A and C. This further leads to UPS impairment and accumulation of toxic ΔK32-lamins A and C (Cattin et al., 2013). UPS impairment has also been demonstrated in cardiomyopathy caused by mutations in desmin or αB-crystallin (CryAB) leading to accumulation of desmin in skeletal and cardiac muscle (Bence et al., 2001; McLendon and Robbins, 2011). A missense mutation of CryAB showed UPS impairment prior to heart hypertrophy and was associated with delivery of ubiquitinated proteins into the 20S proteasome (Chen et al., 2005). Similarly, a mutation in the transcription factor NKX2-5 leads to adult-onset dilated cardiomyopathy and expression of such a specific mutant form of NKX2 in COS-7 cells or HL-1 cardiomyocytes causes UPS impairment (Costa et al., 2013).

Compensatory activation of autophagy might have a role in alleviating the disease pathology after UPS impairment. Our results show that autophagy is enhanced in patient cells in general, as seen by the increased perinuclear accumulation of Atg5 and Atg7, indicating autophagosome formation. Furthermore, lamins A and C were increasingly associated with LC3 in patient cells, suggesting that the enhanced rate of autophagy is, at least partially, due to degradation of lamins A and C. In consistent with our results, others have also shown autophagic degradation of lamins A and C. For example, in Hutchinson-Gilford progeria syndrome (HGPS), the E3 ubiquitin ligase SMURF2 was shown to oligo-ubiquitinate lamins A and C and multi-monoubiquitinate progerin for degradation through autophagy (Borroni et al., 2018; Blank, 2020). Park et al. also showed that in embryonic fibroblasts obtained from the *Lmna* ^H222P/H222P^ mice, a model for Emery-Dreifuss muscular dystrophy, lamins A and C were degraded through autophagy (Park et al., 2009). Interestingly, in a transgenic *Drosophila melanogaster* model mimicking human cardiomyopathy, overexpression of Atg1 eliminated aggregates of mutant lamin C and reduced cardiac arrhythmia, suggesting that enhanced autophagy activity and processing of lamins A and C are beneficial for cell and tissue homeostasis (Bhide et al., 2018).

We further show that lentiviral expression of FLAG-tagged p.S143P lamin A in HeLa cells leads to dysfunctional UPS, indicating that the defect found in patient cells is likely due to mutant lamin. The exact mechanism has yet to be elucidated, but we have previously shown that p.S143P lamins A and C are more nucleoplasmic compared to wild-type lamins A and C, are incapable of forming normal filaments, and occasionally assemble into disorganized aggregates (West et al., 2016) that may be more prone to degradation. Whether it is only the mutant forms of lamins A and C that are K48-ubiquitinated in the heterozygote patient cells and choke up UPS would be of interest to analyze in future studies. For this purpose, a mutant-specific antibody against lamins A and C would be highly beneficial. In agreement with this hypothesis, we have earlier shown that inhibition of UPS using MG132 increases aggregation of lamins A and C in the nucleoplasm of lentivirally transduced fibroblasts expressing p.S143P-LA (West et al., 2016). In the present study, we also show that UPS dysfunction can be reversed by treatment with a chaperone, 4-PBA. The reversibility of UPS activity indicates that the decrease in activity is due to the buildup of degradation products. We found that 4-PBA also enhanced autophagy in both control and patient cells, which further augmented the degradation of proteins. A similar strategy, i.e., activation of autophagy, has also been used in other models of laminopathies, e.g. in the Lmna^H222P/H222P^ mice, where the mTOR pathway inhibitor temsirolimus enhanced autophagy and improved cardiac function (Choi et al., 2012). UPS and autophagy are highly dynamic and quickly adapting systems that preserve cellular homeostasis through interplay. Still, a default in one of the systems can cause severe disease, especially in the long run. Accumulation of misfolded proteins is linked to a variety of different diseases such as neurodegenerative diseases, cancer, diabetes, lysosomal storage diseases and cardiovascular diseases (Amm et al., 2014; Maejima, 2020). Damaged, misfolded, or non-functional proteins are removed through UPS, but impairment or inhibition of UPS leads to protein accumulation within the cell. Aggregated proteins that cannot unfold to pass through the proteolytic barrel in the proteasomes can inhibit UPS and are instead degraded through autophagy (Verhoef et al., 2002). To protect the cells from toxic buildup, the aggregated proteins are transported towards the microtubule-organizing center (MTOC) where the aggresomes are formed (Johnston et al., 1998; Kopito, 2000). Cytoplasmic aggresomes are enriched in chaperones, ubiquitin and proteasomal subunits with a cage of vimentin (Wójcik et al., 1996). In the patient cells, cytoplasmic accumulations of lamins A and C co-localizing with ubiquitin were detected after inhibition of UPS with MG132. These accumulations were surrounded by vimentin such as the aggresomes. Whether they are equal to the aggresomes is unclear; however, similar cytoplasmic accumulations were not detected in untreated cells. Instead, the patient cells showed accumulated K48-linked lamins A and C in the nucleus, which could represent sequestration within the nucleus. Reported sequestration within the nucleus includes cajal bodies, PML bodies, nuclear speckles, and nucleoli (Fu et al., 2005; Park et al., 2011). It is possible that the p.S143P mutant forms of lamins A and C are misfolded, unable to pass through the proteolytic barrel of UPS and further “choking” it up, which leads to sequestration of lamins A and C into compartments for further degradation. Activation of autophagy in patient cells might be an early step in the disease mechanism to eliminate misfolded and damaged proteins. External stresses that burden autophagy could increase misfolded lamins A and C above a critical level that leads to the formation of visible nuclear aggregates. However, the results of the current study were limited to cell cultures derived from one patient and an age-matched control. Whether the UPS is equally dysfunctional in cell cultures obtained from patients with other *LMNA* mutations remains to be analyzed in future studies.

**FIGURE 5:**
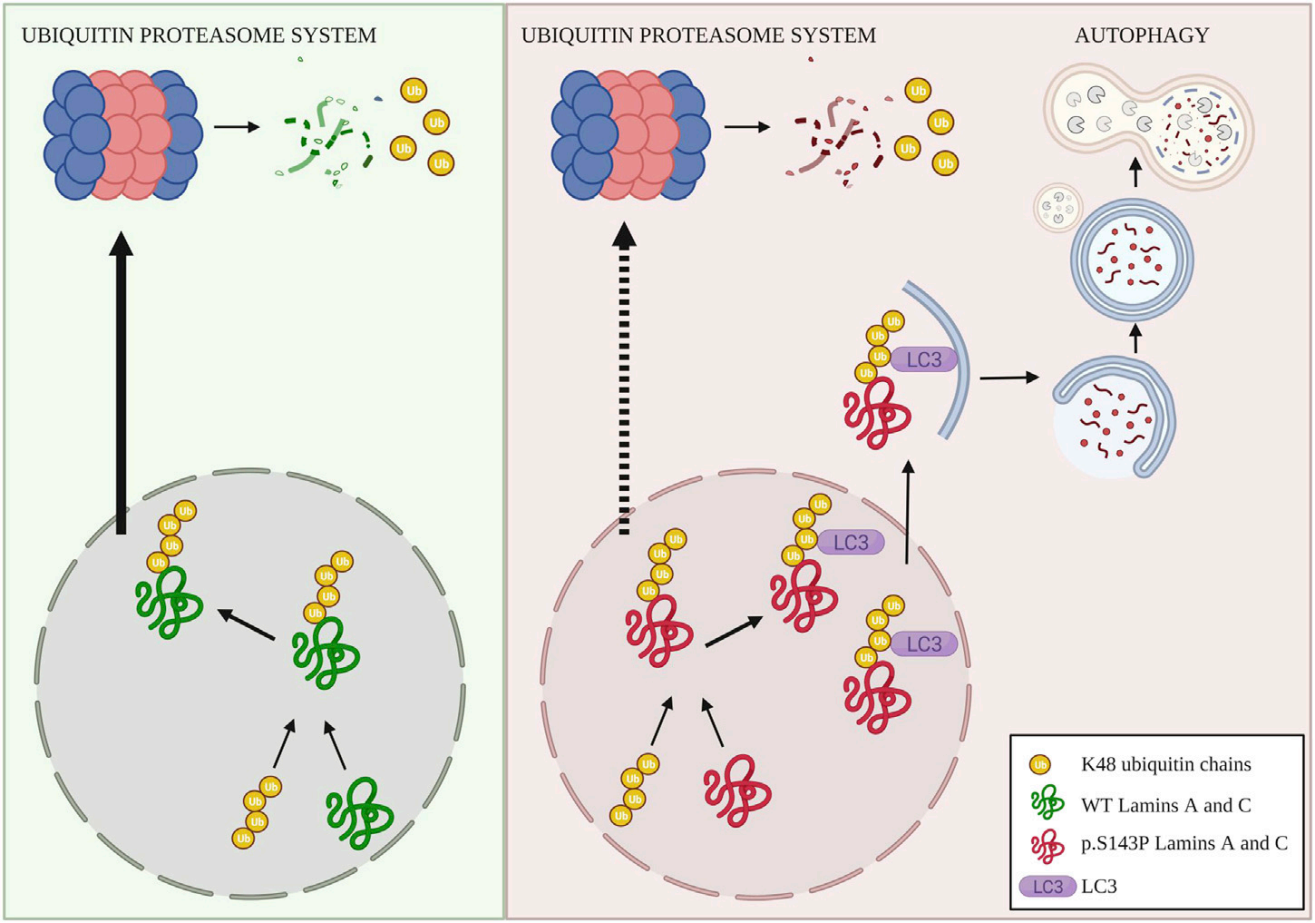
Illustrative picture of degradation of K48-ubiquitinated lamins A and C. In normal cells, wild-type lamins A and C are partially ubiquitinated with K48-linked chains that lead to UPS-mediated degradation of lamins A and C. In the patient cells, lamins A and C are increasingly K48-ubiquitinated, presumably due to increased turnover and production, as well as proteasomal dysfunction. This leads to saturation of UPS and an accumulation of K48-linked ubiquitin chains within the nucleus. Saturation and dysfunction of UPS further lead to compensatory degradation of K48-lamins A and C through autophagy, created with BioRender.com.

In summary, we suggest that in normal cells a small portion of lamins A and C is K48-ubiquitinated and degraded by UPS while in the lamin mutant cells, lamins A and C are increasingly expressed, K48-ubiquitinated and targeted for degradation through UPS (Figure 5). This leads to a dysfunction of UPS and a buildup of K48-ubiquitin chains within the nucleus. Toxic accumulation of ubiquitinated targets could be part of the disease mechanism within the patient tissues, and the ongoing burden of UPS degradation and/or external stresses may eventually increase the impairment of UPS. In the patient cells lamins A and C are degraded by compensatory enhanced autophagy (Figure 5). Whether the patient cells, can uphold enhanced autophagy still remains to be answered. Additionally, autophagy activity has been reported to decrease with age, which would have a tremendous effect on cell homeostasis. Promisingly, a chaperone used in this study, 4-PBA, restored UPS activity and further enhanced autophagy and normalized protein levels of lamins A and C in the patient cells. Such small molecular drugs may eventually turn out to be beneficial for the treatment of laminopathies with similar molecular etiology.

## Data Availability

The mass spectrometry proteomics data have been deposited to the ProteomeXchange Consortium via the PRIDE (Perez-Riverol et al., 2022) partner repository with the dataset identifier PXD033937.

## Supplementary material

Supplementary Figure S1 Lamin A and C staining after NH_4_Cl treatment. **(A)** Pearson’s correlation coefficients of p62 and LC3-I/II stainings as determined from untreated and NH_4_Cl-treated control and patient cells. The data was retrieved from 10 randomly selected confocal images including 60-100 individual cells per sample. Note an increased correlation upon NH_4_Cl treatment. **(B)** Pearson’s correlation coefficients of p62 and lamin A/C stainings as determined from untreated and NH_4_Cl-treated control and patient cells. **(C)** Control and patient cells stained with antibodies against p62 and lamins A and C show slightly increased lamin A and C intensities after treatment with 20 mM NH_4_Cl for 24 h. Scale bar 10 µm. The whiskers show mean values ± s.d **p* < 0.05.

Supplementary Figure S2 Cytoplasmic changes after proteasome inhibition with MG132. **(A)** Acridine orange staining from untreated and MG132-treated control and patient cells showing acidic vesicular organelles (AVO) in red and cell nucleus and membranes in green. **(B)** Treatment of control and patient cells with 1 µM MG132 for 24 h leads to accumulation of cytoplasmic K48 ubiquitin which is surrounded by perinuclear vimentin cage (shown with arrows). Scale bar 10 µm.
