# Supplementary figures and images for: A heterozygous p.S143P mutation in LMNA associates with proteasome dysfunction and enhanced autophagy-mediated degradation of mutant lamins A and C
Source: Front Cell Dev Biol. 2022 Aug 30;10:932983. doi: 10.3389/fcell.2022.932983 (PMC9468711; doi:10.3389/fcell.2022.932983)

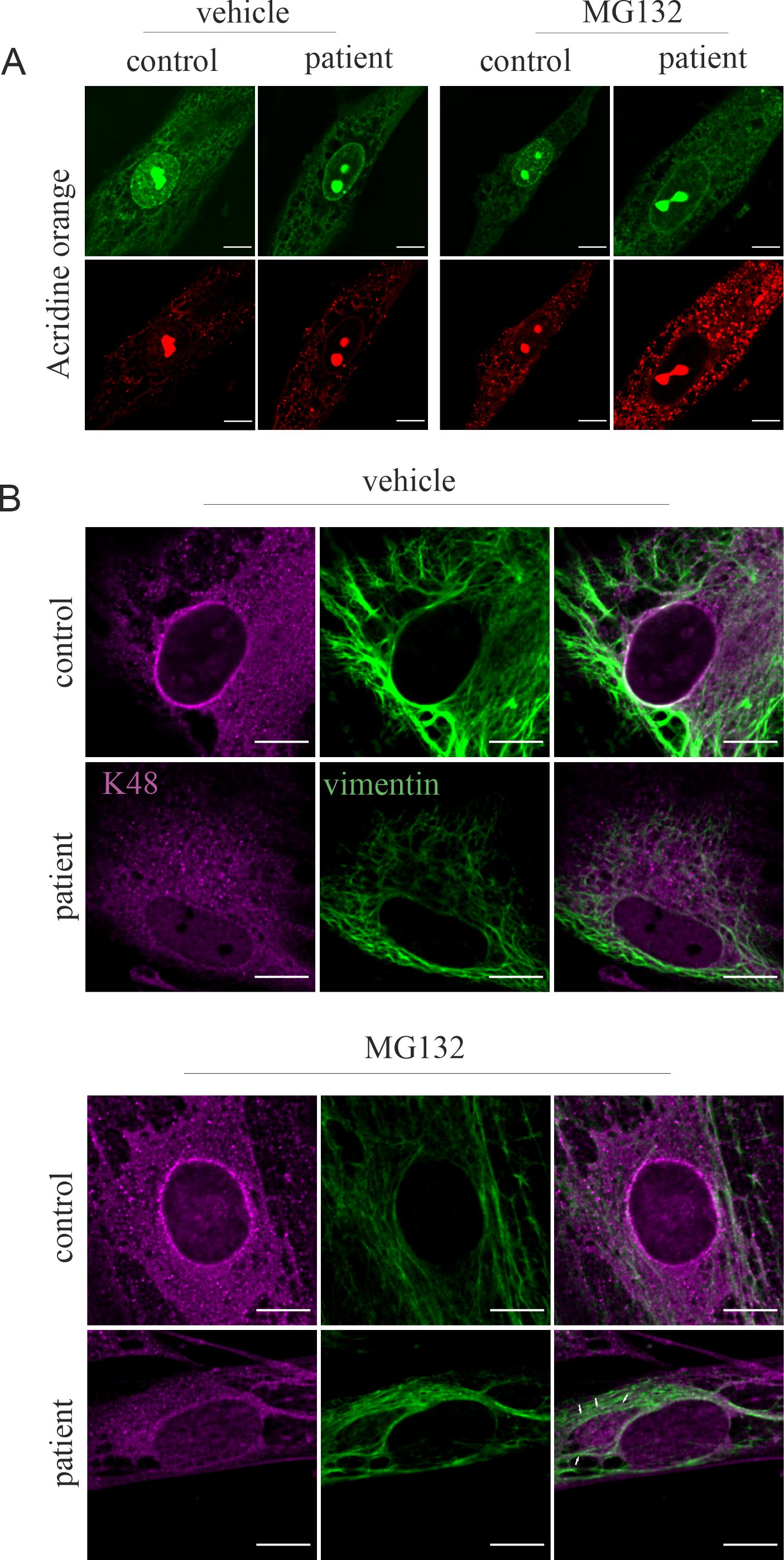

Supplement: Supplementary file 1 [file Image2.jpg]

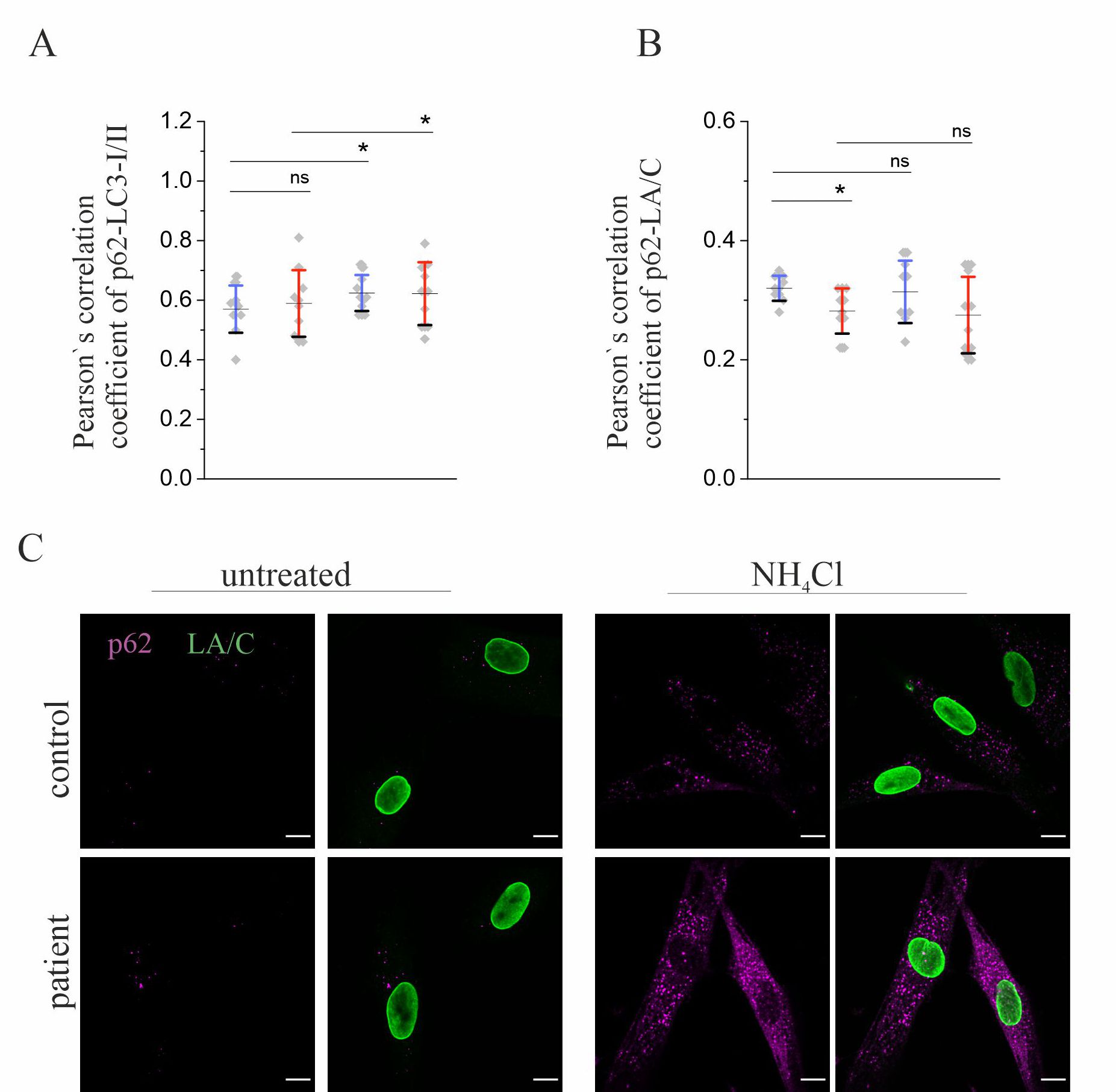

Supplement: Supplementary file 2 [file Image1.jpg]
